# Tranexamic Acid Combined with Compression Bandage Following Total Knee Arthroplasty Promotes Blood Coagulation: A Retrospective Analysis

**DOI:** 10.1155/2020/2739560

**Published:** 2020-11-02

**Authors:** Guo-hua Li, Li-ming Ding, Lei Sun, Fu Wang

**Affiliations:** Department of Joint Surgery, Dong'a People's Hospital, Liaocheng 252200, Shandong Province, China

## Abstract

**Objective:**

This paper included a retrospective review of the effect of tranexamic acid (TXA) combined with pressure bandaging on hemostasis of patients who received a unilateral total knee arthroplasty (TKA) from 2017 to 2019.

**Methods:**

A total of 197 patients undergoing TKA were chosen to be classified into 2 groups, the compression bandage control group and compression bandage combined with TXA observation group. The patients received blood routine examination when they were in the 1st, 3rd, and 6th days of before and postoperation. Some parameters, such as hemoglobin (Hb), C-reactive protein (CRP), D-dimer value, fibrinogen, prothrombin time (PT), activated partial thromboplastin time (APTT), thrombin time (TT), international normalized ratio (INR), and erythrocyte sedimentation rate (ESR), were also investigated.

**Results:**

In our research, the mean age was 66.54 ± 7.95 years. No difference was found in patient sex (*P* = 0.876) and age (*P* = 0.749) between groups. No differences were found in the levels of Hb, fibrinogen, TT, and INR between the 2 groups at each period (*P* > 0.05). The difference of PT was significantly different on the 1st day (*P* = 0.011), 3rd day (*P* = 0.010), and 6th day (*P* = 0.004) after surgery. Besides, the changes in APTT in observation group were clearly higher compared with the control group on the 3rd day (*P* = 0.001) and 6th day (*P* = 0.001). On the 3rd and 6th days after operation, the CRP level of the two groups increased continuously, and the CRP level was significantly higher in the observation group in comparison with the control group (*P* = 0.008, *P* = 0.010). On 1st and 3rd days after surgery, compared to the control group, the D-dimer level of patients in the observation group was distinctly fewer (*P* = 0.001, *P* = 0.027).

**Conclusion:**

TXA combined with compression bandage is a potential option for the reduction of bleeding after TKA.

## 1. Introduction

Total knee arthroplasty (TKA) has always been well known as an effective project for the treatment of advanced osteoarthritis or rheumatoid arthritis. Although it can reduce the pain of patients and improve the knee joint function, it is prone to excessive blood loss, resulting in blood transfusion [1–3]. Blood transfusion may also be recognized to cause a series of adverse reactions, infection, immune response, and myocardial infarction, for example, and to increase the patients' costs [1]. Based on these problems to solve, some tourniquets and antifibrinolytic drugs are most often used to lessen the loss amount of blood volume during the perioperative period [4].

Tranexamic acid (TXA) is a lysine derivative, which is structurally similar to lysine. It can be associated with lysine targets on the plasminogen to help cut the interaction between fibrin and heavy chain of plasmin, thus directly lead to the promotion of coagulation process and the control of postoperative blood loss [4, 5].Furthermore, TXA is often injected intravenously or intra-articularly so as to weaken the perioperative blood loss of TKA [6–9] in many clinical practices. And then, there are also a number of studies that have found that TXA maybe play an important effect on inflammatory response other than reduce blood loss [5, 10, 11]. However, there is no relevant report on the study of the coagulation function and inflammatory response of TXA combined with compression bandage treatment for patients after TKA in the Dong'a People's Hospital of Liaocheng, Shandong Province, from 2017 to 2019.

This study reviewed 197 patients who received unilateral TKA for the first time in the Dong'a People's Hospital of Liaocheng, Shandong Province, from 2017 to 2019. The effects of TXA combined with pressure bandage and single-pressure bandage on the blood coagulation of patients were compared and analyzed.

## 2. Materials and Methods

### 2.1. Participant Information

197 patients who needed unilateral total knee arthroplasty treatment in the Dong'a People's Hospital of Liaocheng (Shandong province, China) were obtained and enrolled in this research from 2017 to 2019. The inclusion criteria in this research are the following: (a) the osteoarthritis of knee joint was clinically diagnosed, and the first unilateral TKA operation was proposed; (b) no thrombus in patient's two lower limbs before operation; and (c) normal hemoglobin (Hb) and coagulation indexes before operation. The exclusion criteria involved that patients had (a) coagulation dysfunction; (b) a history of knee replacement, infection or severe deformation of the knee joint; (c) the TXA or involved drugs allergies in surgery; (d) thrombus of both lower limbs indicated by the preoperative color Doppler ultrasound; and (e) could not tolerate the operation due to poor physical function.

All the patient specimens with their families have informed and signed the informed consent. Meanwhile, the study was approved by the ethics committee of our hospital (registration No.: ChiCTR2000033255).

### 2.2. Patients Grouping

All patients in 2017 (*n* = 36) underwent only the pressure dressing after surgery, as the control group. In 2018, 75 patients were blindly distributed and randomly grouped into the following: control group, patients (*n* = 26) who received only a pressure dressing after surgery, and observation group, patients (*n* = 49) who received compression bandage combined with tranexamic acid after surgery. And in 2019, 86 patients were randomly allocated into two groups in the above same method: control group, patients (*n* = 59) who were treated only with pressure dressing after surgery, and observation group, patients (*n* = 27) who were treated with compression bandage combined with tranexamic acid. All perioperative managements of TKA were operated according to a well-established multimodal enhanced-recovery strategy.

### 2.3. Procedures

In this retrospective study, all patient samples were perioperatively treated with general anesthesia and standard analgesia. They were routinely treated by using the preoperative tourniquet, and also, the systolic blood pressure was about 100 mm Hg. Subsequently, combined spinal anesthesia (CSEA) was carried out. The patients were operated by a standard medial parapatellar approach and kept the knees bent to reduce bleeding during the operation. Then, the knee prosthesis (AKKnee, AK Medical International Limited, Beijing, China) was installed, the synovium was not removed, and the patella surface was trimmed, but not replaced. In the control group, the wound was sutured directly after careful hemostasis and was protected by a cotton pad and wrapped with an elastic bandage with pressure of 300 mm Hg. Furthermore, on the basis of pressure bandaging, the corresponding dose of tranexamic acid (diluted with 0.9% sodium chloride solution) was injected intravenously into the patients in the observation group. For details, please refer to the Figure S1.

### 2.4. Postoperative Management

All patients were not treated with the intra-articular drainage tube. Within 24 hours after the surgery, the low-flow oxygen inhalation, electrocardiographic monitoring, blood oxygen saturation, and blood pressure were routinely monitored and observed. Intermittent cold compresses were applied around the incision for 48 hours. Then, these patients were subcutaneously given 0.4 mL/d of low molecular weight heparin sodium 12 h after surgery, and this lasted until 10 days after surgery. The blood transfusion was needed if someone had the following conditions: hemoglobin < 70 g/L or some anemia symptoms such as dizziness, palpitation, and mental malaise appeared although hemoglobin was 70-100 g/L.

### 2.5. The Indicators Observated

Patients all needed to receive blood routine examinations before and postoperation of the 1st, 3rd, and 6th days. There were some indicators to record such as Hb, CRP, D-dimer value, fibrinogen, PT, APTT, TT, INR, and ESR.

### 2.6. Statistical Analysis

The SPSS 20.0 (SPSS Inc., Chicago, IL) statistical software was exerted to analyze the data collected in the experiment, and they are presented as the mean ± standard deviation (SD). The generalized estimating equations method was utilized, and *t* test was used for comparison between the two groups. *P* < 0.05 was determined statistically significant.

## 3. Results

### 3.1. The Comparison of Two Groups of General Data and Hb

As shown in Table 1, among the 197 patients, there were 112 cases in the control group with 66.70 ± 7.63 years, and there were 85 cases in the observation group with 66.33 ± 8.38 years. No difference was found in patient sex (*P* = 0.876) and age (*P* = 0.749) between groups. Before surgery, the Hb values of the two groups were 129.71 ± 13.80 and 130.50 ± 13.37, respectively, and there was no marked difference between the two groups (*P* = 0.972). After operation, the Hb showed a decreasing trend in both groups, and there was no significant difference at 1st, 3rd, and 6th days between the two groups (*P* = 0.946, 0.168, 0.271).

### 3.2. The Comparison of Two Groups of CRP and D-Dimer Levels

The values of CRP and D-dimer were reported. From Table 2, there was no significant difference found before surgery in the above two levels between the control group and the observation group (*P* = 0.154, *P* = 0.087). At the 1st day after operation, the levels of the above two indicators were both clearly increased in the two groups, but there was no significant difference in C-reactive levels (*P* = 0.175), a remarkable difference in D-dimer levels (*P* = 0.001) between the two groups. At 3rd day after the operation, the CRP levels of the two groups continued to increase, and the level of CRP in the observation group showed a significantly higher increase compared to the control group (*P* = 0.008). However, the level of D-dimer showed a downward trend, and the D-dimer level of patients in the observation group was largely lower than that of the control group (*P* = 0.027). At the 6th day after surgery, the level of CRP in the observation group was still significantly higher as compared to the control group (*P* = 0.010). Nevertheless, there was no obvious difference between the two groups (*P* = 0.190).

### 3.3. The Comparison of Two Groups of ESR and Fibrinogen Levels

The descriptive values and comparison results of ESR and fibrinogen levels are given in Table 3. It was found that there was no difference before the operation and at 1st day and 3rd day after operation in ESR levels (*P* = 0.703, *P* = 0.084, *P* = 0.021), while a significant difference was shown at 6th day after operation in ESR levels (*P* = 0.003). For the levels of fibrinogen, there was no distinct differences between the two groups in each period (*P* = 0.070, *P* = 0.257, *P* = 0.080, *P* = 0.387).

### 3.4. The Comparison of Two Groups of TT and INR

The descriptive values along with the comparison results of PT and INR were given in Table 4. As exhibited in Table 4, there was no distinct difference in preoperative PT before the operation (*P* = 0.142), and there was a distinct gap between the two groups at 1st day, 3rd day, and 6th day after operation (*P* = 0.011, *P* = 0.010, *P* = 0.004). But for INR, there was no statistically significant differences between the two groups in each period (*P* = 0.744, *P* = 0.414, *P* = 0.153, *P* = 0.081).

### 3.5. The Comparison of Two Groups of APTT and TT

The descriptive values and comparison results of APTT and TT are given in Table 5. Table 5 indicated that no apparent difference existed in preoperative APTT between the two groups before operation and one day after operation (*P* = 0.078, *P* = 0.255), but sharp difference existed between the two groups at 3rd day and 6th day after operation (*P* = 0.001, *P* = 0.001). For TT, there was no marked difference between the two groups before and after surgery (*P* = 0.068, *P* = 0.260, *P* = 0.450, *P* = 0.456). The thrombin time after operation was lower than that before operation.

## 4. Discussion

The percentage of males was close to 30% (male: 29.95%, female: 70.05%) and mean age was 66.54 ± 7.95 years in our study. According to a previous report, the average age of TKA for primary osteoarthritis was 65.14 ± 4.06 years [12]. During the TKA operation, 800-1800 mL of perioperative blood loss may occur during the period despite the use of tourniquets and gel ice [13]. In recent studies, many evidences have shown the beneficial effects of TXA in lowering blood loss in orthopedic surgeries, which plays a big role in inflammatory response [5, 6, 9]. Karaduman et al. evaluated the hematological parameters with TKA between the conventional method such as ice packs and cryotherapy, and they found that postoperative cryotherapy might be a potential, simple, and noninvasive option for TKA patients [13]. In this retrospective analysis, there was no significant difference between the control group and observation group in each period on Hb, fibrinogen, TT, and INR levels (*P* > 0.05), which suggested TXA does not significantly increase the risk of coagulation. These results were consistent with previous reports. In previous reports, intravenous TXA can increase the postoperative Hb level of patients with TKA, reduces the trend of postoperative blood loss, and does not cause thrombus [14, 15].

In patients with arthritis, the coagulation system in synovium of inflamed joints is strongly activated, resulting in fibrin deposition and erythrocyte sedimentation rate increase. As known, fibrinogen (soluble fibrin) is as a precursor of fibrin. It can turn into insoluble fibrin in the final stage of coagulation, which makes blood coagulate [16]. INR is the ISI power of the ratio of prothrombin time of patients to normal control prothrombin time, which can be used to correct the differences in thrombin reagents and standardize the measurement of prothrombin time. After surgery, INR < 1.5 is recommended, which is widely used in the world to reflect the coagulation time [17]. APTT mainly indicates whether the endogenous coagulation is normal. If APTT is shortened, it indicates that the blood is in the increase status of blood coagulation [18]. In our study, it was found that the difference of PT was significantly different on the 1st day (*P* = 0.011), 3rd day (*P* = 0.010), and 6th day (*P* = 0.004) after surgery. Besides, the changes in APTT in the observation group was clearly higher than that of the control group on the 3rd day (*P* = 0.001) and 6th day (*P* = 0.001). Although TXA combined with compression bandage can significantly increase the PT and APTT levels of patients after unilateral TKA compared with the only pressure dressing treatment, INR level showed no obvious difference between the two groups (*P* > 0.05), and the INR values of both groups were less than 1.5. Fortunately, Hb and fibrinogen levels also showed no significant differences between the control group and observation group in each period (*P* > 0.05). These data suggested that TXA-combined compression dressing could alleviate postoperative anemia in TKA patients and promoted blood clotting without causing complications such as thrombus.

In TKA, CRP and D-dimer were determined to evaluate the presence of inflammation [5, 19]. For the role of TXA on inflammatory response, it remains controversial in TKA. Grant et al. found that TXA significantly increased the expression of inflammatory markers after bone cut in contrast to non-TXA patients [20]. Nevertheless, Wu et al. demonstrated that the lower levels of CRP and D-dimer were observed in patients with TXA [5]. In this research, the expression of CRP and D-dimer were obviously increased after surgery. In addition, the levels of CRP were higher in patients with TXA administration compared with non-TXA patients, but the levels of D-dimer were lower in patients with TXA administration. D-dimer's, as a marker of specific fibrinolysis process, concentration change is related to hemostasis. A high level of D-dimer can be used as a marker of thrombus [21]. These data further indicated that TXA might be a safe and effective option to stop the bleeding in the first unilateral TKA operation.

Although this study systematically reviewed and compared the effects of TXA-combined compression dressing and only compression dressing on the coagulation function of patients after TKA surgery, there were some limitations. The postoperative complications, long-term biochemical indexes and HSS scores of patients, were not studied, and the two groups will be followed up in the later period.

## 5. Conclusion

To summarise, we evaluated the efficacy of TXA combined with compression bandage after TKA and found that it is a good choice to only have compression bandage. TXA combined with compression bandage is a potential option for the reduction of bleeding after total knee arthroplasty.

## Figures and Tables

**Table 1:** General information and hemoglobin values of the two groups ($\bar{X}\pm \text{SD}$).

	Control	Observation	*P*
Female	79	59	0.876
Male	33	26	
Age	66.70 ± 7.63	66.33 ± 8.38	0.749
	Hemoglobin(Hb, g/L)		
Preoperation	129.71 ± 13.80	130.50 ± 13.37	0.972
1st day	122.19 ± 13.33	122.26 ± 13.18	0.946
3rd day	110.85 ± 13.65	113.56 ± 13.42	0.168
6th day	112.19 ± 12.18	114.23 ± 13.49	0.271

**Table 2:** Comparison of C-reactive protein and D-dimer levels between the two groups ($\bar{X}\pm \text{SD}$).

Time	C-reactive protein (CRP, mg/L)			D-dimer (mg/L)		
	Control	Observation	*P*	Control	Observation	*P*
Preoperation	5.94 ± 12.75	3.81 ± 5.67	0.154	0.61 ± 1.12	0.39 ± 0.43	0.087
1st day	35.67 ± 23.11	40.72 ± 28.52	0.175	4.79 ± 4.64	2.90 ± 2.51	0.001
3rd day	71.02 ± 45.38	90.87 ± 57.33	0.008	1.85 ± 1.48	1.42 ± 1.10	0.027
6th day	32.02 ± 33.57	47.27 ± 47.83	0.010	2.97 ± 2.06	2.62 ± 1.57	0.190

**Table 3:** Comparison of ESR and fibrinogen levels between the two groups ($\bar{X}\pm \text{SD}$).

Time	Erythrocyte sedimentation rate (ESR, mm/h)			Fibrinogen (g/L)		
	Control	Observation	*P*	Control	Observation	*P*
Preoperation	25.21 ± 19.09	24.04 ± 19.92	0.703	2.89 ± 0.66	2.72 ± 0.58	0.070
1st day	37.07 ± 23.95	43.22 ± 24.30	0.084	3.15 ± 1.33	2.97 ± 0.68	0.257
3rd day	63.17 ± 26.62	72.26 ± 26.25	0.021	4.57 ± 1.00	4.86 ± 1.19	0.080
6th day	62.96 ± 27.55	75.84 ± 30.40	0.003	4.50 ± 1.30	4.65 ± 1.06	0.387

**Table 4:** Comparison of prothrombin time and international normalized ratio between the two groups ($\bar{X}\pm \text{SD}$).

Time	Prothrombin time (PT, s)			International normalized ratio (INR)		
	Control	Observation	*P*	Control	Observation	*P*
Preoperation	11.62 ± 1.35	11.87 ± 0.85	0.142	1.00 ± 0.06	1.00 ± 0.07	0.744
1st day	12.18 ± 1.00	12.56 ± 1.01	0.011	1.27 ± 2.38	1.06 ± 0.08	0.414
3rd day	11.85 ± 0.91	12.19 ± 0.90	0.010	1.01 ± 0.07	1.03 ± 0.07	0.153
6th day	11.97 ± 0.88	12.35 ± 0.90	0.004	1.03 ± 0.07	1.05 ± 0.07	0.081

**Table 5:** Comparison of activated partial thromboplastin time and thrombin time between the two groups ($\bar{X}\pm \text{SD}$).

Time	Activated partial thromboplastin time (APTT, s)			Thrombin time (TT, s)		
	Control	Observation	*P*	Control	Observation	*P*
Preoperation	27.09 ± 3.94	28.85 ± 3.27	0.078	17.30 ± 1.63	17.80 ± 2.17	0.068
1st day	27.62 ± 3.42	28.27 ± 4.39	0.255	16.73 ± 1.03	16.96 ± 1.79	0.260
3rd day	28.67 ± 3.64	30.59 ± 4.07	0.001	15.42 ± 1.03	15.26 ± 1.89	0.450
6th day	27.85 ± 4.78	30.31 ± 4.92	0.001	15.59 ± 1.15	15.39 ± 2.42	0.456

## Data Availability

The data used to support the findings of this study are included within the supplementary information files.

## Supplementary Materials

Supplementary Materials Figure S1 In 2017, there were 36 patients, aged 67.56 ± 6.78, including 11 males and 26 females. All patients in 2017 underwent pressure dressing after surgery without TXA. The indicators were collected before and postoperation of the 1st, 3rd, and 6th days, including Hb, CRP, D-dimer value, fibrinogen, PT, APTT, TT, INR, and ESR. In 2018, there were 75 patients, aged 65.64 ± 8.26, including 16 males and 59 females. Twenty-six patients received pressure dressing after surgery without TXA. Forty-nine patients received compression bandage combined with TXA after surgery. The indicators were collected before and post-operation of the 1st, 3rd, and 6th days, including Hb, CRP, D-dimer value, fibrinogen, PT, APTT, TT, INR, and ESR. In 2019, there were 86 patients, aged 86.64 ± 8.26, including 31 males and 55 females. Fifty-nine patients were treated with pressure dressing without TXA after surgery. Twenty-seven patients were treated with compression bandage combined with TXA. The indicators were collected before and postoperation of the 1st, 3rd, and 6th days, including Hb, CRP, D-dimer value, fibrinogen, PT, APTT, TT, INR, and ESR.
