# Supplementary figures and images for: Tranexamic Acid Combined with Compression Bandage Following Total Knee Arthroplasty Promotes Blood Coagulation: A Retrospective Analysis
Source: Biomed Res Int. 2020 Nov 2;2020:2739560. doi: 10.1155/2020/2739560 (PMC7655242; doi:10.1155/2020/2739560)

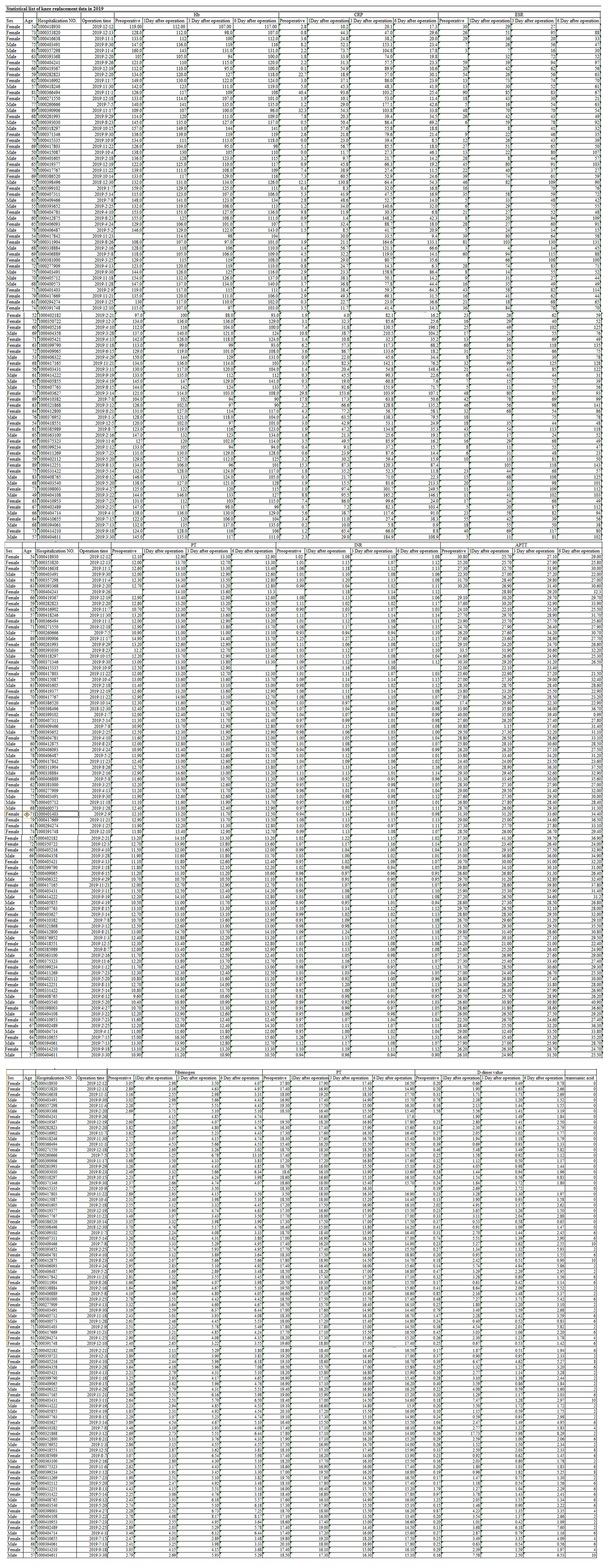

Supplement: Supplementary Materials — Figure S1 In 2017, there were 36 patients, aged 67.56 ± 6.78, including 11 males and 26 females. All patients in 2017 underwent pressure dressing after surgery without TXA. The indicators were collected before and postoperation of the 1st, 3rd, and 6th days, including Hb, CRP, D-dimer value, fibrinogen, PT, APTT, TT, INR, and ESR. In 2018, there were 75 patients, aged 65.64 ± 8.26, including 16 males and 59 females. Twenty-six patients received pressure dressing after surgery without TXA. Forty-nine patients received compression bandage combined with TXA after surgery. The indicators were collected before and post-operation of the 1st, 3rd, and 6th days, including Hb, CRP, D-dimer value, fibrinogen, PT, APTT, TT, INR, and ESR. In 2019, there were 86 patients, aged 86.64 ± 8.26, including 31 males and 55 females. Fifty-nine patients were treated with pressure dressing without TXA after surgery. Twenty-seven patients were treated with compression bandage combined with TXA. The indicators were collected before and postoperation of the 1st, 3rd, and 6th days, including Hb, CRP, D-dimer value, fibrinogen, PT, APTT, TT, INR, and ESR. [file 2739560.f1.zip › 2019 (1).jpg]
